# Pediatric Lemierre’s Syndrome: A Comprehensive Literature Review

**DOI:** 10.3390/pediatric16010018

**Published:** 2024-03-18

**Authors:** Salvatore Lavalle, Edoardo Masiello, Salvatore Cocuzza, Piero Pavone, Alessandra Di Nora, Christian Calvo-Henriquez, Jerome Rene Lechien, Miguel Mayo Yanez, Andrea Praticò, Manuela Ceccarelli, Giannicola Iannella, Annalisa Pace, Federica Maria Parisi, Giuseppe Magliulo, Antonino Maniaci

**Affiliations:** 1 Faculty of Medicine and Surgery, University of Enna Kore, 94100 Enna, Italy; salvatore.lavalle@unikore.it (S.L.); andrea.pratico@unikore.it (A.P.); manuela.ceccarelli@unikore.it (M.C.); 2 Radiology Unit, Department Clinical and Experimental, Experimental Imaging Center, Vita-Salute San Raffaele University, 20132 Milan, Italy; 3 Department of Medical, Surgical Sciences and Advanced Technologies “GF Ingrassia” ENT Section, University of Catania, 95123 Catania, Italy; s.cocuzza@unict.it (S.C.); federicamariaparisi@gmail.com (F.M.P.); 4 Department of Child and Experimental Medicine, Section of Pediatrics and Child Neuropsychiatry, University of Catania, 95100 Catania, Italy; ppavone@unict.it (P.P.); alessandradinora@gmail.com (A.D.N.); 5 Service of Otolaryngology, Hospital Complex of Santiago de Compostela, 15701 Santiago de Compostela, Spain; christian.calvo.henriquez@gmail.com; 6 Head and Neck Study Group of Young-Otolaryngologists of the International Federations of Oto-Rhino-Laryngological Societies (YO-IFOS), 75000 Paris, France; jerome.lechien@umons.ac.be (J.R.L.); miguelmmy@gmail.com (M.M.Y.); 7 Department of Anatomy and Experimental Oncology, Mons School of Medicine, University of MONS, 7022 Mons, Belgium; 8 Department of Otorhinolaryngology-Head and Neck Surgery, Complexo Hospitalario Universitario A Coruña (CHUAC), 15006 A Coruña, Spain; 9 Department of ‘Organi di Senso’, University “Sapienza”, Viale dell’Università, 33, 00185 Rome, Italy; giannicola.iannella@uniroma1.it (G.I.); annalisa.pace@uniroma1.it (A.P.); giuseppe.magliulo@uniroma1.it (G.M.)

**Keywords:** Lemierre’s syndrome, neck abscess, internal jugular vein thrombophlebitis, septic thrombophlebitis, pediatric

## Abstract

Background: Lemierre syndrome is a rare, potentially fatal complication of oropharyngeal infections characterized by septic thrombophlebitis of the internal jugular vein. It primarily affects healthy adolescents and young adults. Its incidence declined after the antibiotic era, but it may have resurged in recent decades, likely due to judicious antibiotic use and increasing bacterial resistance. Prompt diagnosis and treatment are imperative to prevent significant morbidity and mortality. Methods: Lemierre syndrome has been called “the forgotten disease,” with a reported incidence of around 3.6 cases per million. The mean age at presentation is around 20 years old, though it can occur at any age. Lemierre Syndrome follows an oropharyngeal infection, most commonly pharyngitis, leading to septic thrombophlebitis of the internal jugular vein. *F. necrophorum* is the classic pathogen, though other organisms are being increasingly isolated. Metastatic infections, especially pulmonary, are common complications. Contrast-enhanced CT of the neck confirming internal jugular vein thrombosis is the gold standard for diagnosis. Long-course broad-spectrum IV antibiotics covering anaerobes are the mainstays of the disease’s treatment. Anticoagulation may also be considered. Mortality rates are high without treatment, but most patients recover fully with appropriate therapy. Conclusions: Lemierre syndrome should be suspected in patients with prolonged pharyngitis followed by unilateral neck swelling and fevers. Early diagnosis and prompt antibiotic therapy are key, given the potential for disastrous outcomes if untreated. An increased awareness of Lemierre syndrome facilitates its timely management.

## 1. Introduction

Lemierre’s syndrome is a rare, potentially life-threatening condition characterized by septic thrombophlebitis of the internal jugular vein following an oropharyngeal infection. It has been dubbed “the forgotten disease [1]” due to its decline in incidence after the antibiotic era, though it may have resurged in recent decades as judicious antibiotic use has led to increased bacterial resistance. Lemierre’s syndrome was first described in 1900 by Courmount and Cade, who reported a case of suppurative thrombophlebitis of the internal jugular vein associated with oropharyngeal infection. In 1936, Andre Lemierre published a case series of 20 patients with “postanginal septicemia”, linking the disease to the anaerobic pathogen *F. necrophorum* [2]. Lemierre’s syndrome classically follows acute oropharyngitis leading to septic thrombophlebitis of the internal jugular vein (IJV), with secondary septic embolization to distant sites. After introducing antibiotics, especially penicillin, Lemierre’s syndrome’s reported incidence declined drastically. However, over the past two decades, there has been a resurgence in the interest and reporting of Lemierre’s syndrome. If it reflects a true epidemiological trend, this re-emergence will be considered multifactorial, attributed to more judicious antibiotic prescribing practices for pharyngitis leading to increased bacterial resistance, improved anaerobic culturing techniques, and enhanced provider awareness of the disease [3]. The incidence is estimated to be at around 1 per 100,000 among children and young adults. Lemierre’s syndrome most commonly affects previously healthy adolescents and young adults, with a slight male predominance [4]. The average age of onset is around 21 years old, though Lemierre’s syndrome can affect patients of any age, including young children. Mortality remains substantial at 2–4% despite antibiotic availability, underscoring the need for prompt recognition and treatment [5]. The pathogenesis of Lemierre’s syndrome begins with an oropharyngeal infection that spreads from the pharyngeal mucosa to invade the lateral pharyngeal space and carotid sheath. Direct vessel wall invasion or septic emboli lead to suppurative thrombophlebitis of the internal jugular vein. Dissemination of septic emboli can then occur, most often metastasizing to the lungs and joints. Multiple factors are thought to predispose patients to develop this severe sequela of oropharyngeal infections, including glucocorticoid use, immunodeficiency, and malnutrition. *F. necrophorum* expresses multiple virulence factors that allow for platelet aggregation and the evasion of host defenses. Other organisms, such as *streptococci, staphylococci*, and *peptostreptococci*, may also be implicated [6]. Given the potential for disastrous outcomes, including sepsis, multiorgan failure, and death, the prompt recognition of Lemierre’s syndrome based on clinical presentation, followed by targeted diagnostic testing and the immediate initiation of broad-spectrum antibiotics, is imperative. Increasing the awareness of Lemierre’s syndrome amongst providers is key to facilitating rapid diagnosis and lifesaving treatment. Thus, we performed a comprehensive review of Lemierre’s syndrome to consolidate the current knowledge of its epidemiology, pathogenesis, clinical manifestations, and treatment strategies, thereby enhancing the early recognition and management of this life-threatening condition. This review also aimed to highlight the latest research findings, including the emergence of antibiotic-resistant strains, and to provide evidence-based recommendations to improve patient outcomes.

## 2. Materials and Methods

A comprehensive review of the existing literature was conducted using PubMed, Embase, the Cochrane Library, and the Scopus database to study Lemierre’s syndrome in children. The search concepts included the following: Lemierre’s syndrome OR Lemierre’s disease OR necrobacillosis OR postanginal septicemia, Pediatric OR pediatric OR child OR children OR infant OR adolescent, Internal jugular vein thrombosis OR oropharyngeal infection. Our searches were limited to English-language publications. The references of included studies were hand-searched. Two independent reviewers screened the titles, abstracts, and full texts of retrieved records to identify studies meeting pre-defined eligibility criteria. Disagreements were resolved through discussion. Data extracted included medical history, diagnostic workup and imaging, pathogenic organisms, treatment provided, clinical course and outcomes, and morbidity and mortality rates. Consequently, according to AMSTAR-2 guidelines, we formulated 10 research questions to better describe each disease aspect: (1) What is the typical presentation of Lemierre’s syndrome in children? (2) What organisms commonly cause Lemierre’s syndrome? (3) What imaging modalities are recommended for diagnosis? (4) What is the diagnostic accuracy of CT vs. MRI? (5) What treatment regimens are most used? (6) What complications and metastatic foci occur in pediatric Lemierre’s syndrome? (7) What are the common morbidity and mortality rates with appropriate treatment? (8) What risk factors predispose children to developing Lemierre’s syndrome? (9) How do the pathogenesis, presentation, and management differ between children and adults? (10) What are the long-term outcomes for pediatric patients? Observational studies, including prospective and retrospective cohort studies, case–control studies, and case series were all included, as were experimental study designs, including randomized controlled trials. Case reports, conference abstracts, editorials, and narrative reviews were excluded.

### 2.1. What Is the Typical Presentation of Lemierre’s Syndrome in Children?

**Figure 1:**
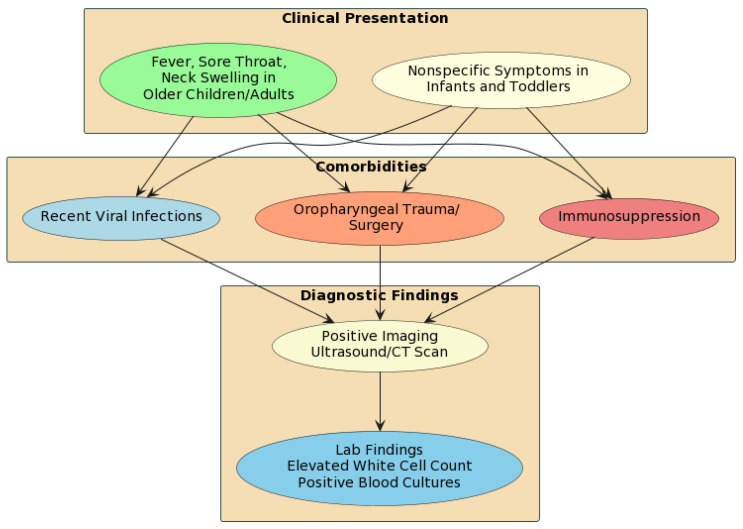
Flow diagram of clinical and diagnostic Lemierre syndrome presentation.

The typical presentation of Lemierre’s syndrome in children involves persistent pharyngitis or tonsillitis followed by spiking fevers, neck pain, and unilateral neck swelling. Additional symptoms may include a sore throat, dysphagia, dyspnea, and cough (Figure 1).

On exam, unilateral cervical lymphadenopathy and tenderness may be noted. Children over 2 years old tend to present similarly to adults. However, infants under 2 years old may present more nonspecifically with irritability, respiratory distress, or sepsis syndrome [5].

### 2.2. What Organisms Commonly Cause Lemierre’s Syndrome?

The most common infectious agent for LS is *F. necrophorum*, and the second most common cause of LS is *F. nucleatum* [7]. The precise mechanism by which *F. necrophorum* induces Lemierre’s syndrome remains uncertain. One proposed hypothesis suggests a scenario of co-infection, where an initial invasive pathogen causes localized mucosal damage and inflammation, facilitating the establishment of an infection with *F. necrophorum* [8]. Once established, it is theorized that *F. necrophorum* may disseminate to the internal jugular vein (IJV) through the tonsillar vein, the lymphatic system, or through the direct invasion and attachment of peritonsillar abscesses to veins within the loose connective tissue of the pharynx [9]. Various bacteria have been identified, although at significantly lower rates. The *S. anginosus* group (SAG) typically colonizes the reproductive and digestive tracts, as well as the respiratory cavity, and has the potential to cause visceral suppurative infections. The SAG is notable for its propensity to form abscesses and empyema. However, determining the causal role of these bacteria in a particular infection can be challenging due to their presence as normal flora in the oral cavity and respiratory tract [10]. While a limited number of Lemierre syndrome cases involving SAG species have been reported, they appear to represent an infrequent group of pathogens in this syndrome [11]. *F. necrophorum* is the classic causative pathogen, being identified in over 80% of pediatric cases in some series. However, other organisms have been increasingly identified as causative, including streptococcal and staphylococcal species and anaerobes like *peptostreptococcus*. Polymicrobial infections have been reported in up to 18% of cases [8].

### 2.3. What Imaging Modalities Are Recommended for Diagnosis?

Diagnosis is often based on a combination of clinical and laboratory findings. However, initial clues for diagnosis are frequently derived from radiological findings, such as the presence of internal jugular vein (IJV) thrombophlebitis, preceding the identification of positive bacterial growth in cultures. Imaging studies are crucial for visualizing thrombophlebitis and abscess formation in the neck. Despite lacking specific guidelines from the literature, ultrasound (US) and chest X-rays have been suggested as initial diagnostic imaging methods [12]. Many cases present in the Emergency Department, with the identification of IJV thrombophlebitis often serving as the primary concrete indication of Lemierre syndrome in numerous patients [13]. In the US, internal jugular vein (IJV) thrombophlebitis may manifest as either a hyperechoic or hypoechoic mass, which is indicative of a thrombus within the vessel. Color Doppler and spectral Doppler imaging are utilized to assess blood flow and detect abnormalities or obstructions due to the thrombus [6]. They can also demonstrate the absence of normal respiratory phasicity and cardiac pulsatility. However, as cited in the literature, ultrasonography is known to have a lower diagnostic accuracy when compared to computed tomography (CT) and magnetic resonance imaging (MRI) [10,11,12,13]. This includes lower sensitivity and specificity, as well as challenges in detecting thrombi that are located beneath the clavicle, the involvement of infectious processes in the mandible, or in visualizing newly formed clots, which often exhibit poor intrinsic echogenicity. It is well established that detecting jugular vein thrombosis is not particularly sensitive or specific to Lemierre’s syndrome. This is due, in part, to the fact that clinical manifestations of Lemierre’s syndrome may arise from a septic thrombotic focus located in veins other than the jugular, which are smaller and less conspicuous [3]. Moreover, when patients seek medical care, the thrombus in the jugular vein might have already resolved or diminished in size, making it less detectable through clinical examination or even imaging studies [6,7]. Therefore, clinicians must maintain a high index of suspicion and consider the overall clinical context when evaluating patients who may have Lemierre’s syndrome. Given these limitations, while ultrasonography can provide supplementary information, it primarily confirms the presence of pre-existing clots [14]. It is crucial, however, to acknowledge that the perceived limitations of ultrasound in evaluating the internal jugular vein (IJV) for thrombophlebitis may be attributed to an operator’s lack of experience with the proper techniques for examining these vessels. While technically demanding, the process can yield high accuracy when performed correctly, as the skill involved is operator-dependent. Scanning these veins is not only feasible but can provide precise information. It is essential not to rely solely on the B-mode scan, which might not always reveal a fresh thrombus due to its limitations. A critical aspect of the ultrasound examination is assessing the vein’s collapsibility, which is notably reduced in the presence of a thrombus. To mitigate the risk of dislodging a clot during this compression maneuver, alternative patient positions, such as sitting or lateral decubitus, can be employed [15]. This approach allows for the examination of the IJV without the need for direct compression, potentially providing a safer and more effective method for detecting thrombi. The preferred diagnostic method for Lemierre syndrome is a contrast-enhanced CT scan of the neck and chest. This imaging technique is optimal for depicting the site of primary infection and revealing characteristic features like a filling defect in the IJV, variably associated with edema in adjacent tissues [16]. It can also identify complications such as osteomyelitis, arthritis, abscesses, and septic pulmonary emboli, providing information about their extent and distribution within the lungs. Typically, the latter exhibits a classical pattern, appearing as multiple peripherals, round, wedge-shaped areas that evolve into cavities. Cavitation is observed as peripheral enhancement with diminished central attenuation, often accompanied by a discernible feeding blood vessel entering the lesion—a feature known as the “feeding vessel sign”, observed in approximately two-thirds of cases [17]. A CT scan also be helpful in distinguishing atypical cases of Lemierre’s syndrome [18]. Indeed, reported instances have highlighted thrombosis occurring in the facial vein rather than the IJV [19,20]. A retrospective study examining 11 cases of Lemierre’s syndrome revealed that 4 out of 11 exhibited thrombosis in the tributaries of the IJV [21]. The radiological triad pharyngitis, cervical vein thrombosis, and cavitating pulmonary lesions are typical of this syndrome [22]. Thus, the radiologist may be the first to recognize it and have a key role in this still high-mortality pathology.

### 2.4. What Is the Diagnostic Accuracy of CT vs. MRI?

Direct comparative studies still need to be conducted. Both CT and MRI demonstrate high accuracy in detecting the hallmark of IJV thrombosis with sensitivity [23]. Unavoidably, CT scans entail exposure to ionizing radiation and involve the administration of an iodine-containing contrast medium. MRI has a heightened sensitivity to slow flow rates, superior soft-tissue contrast, and the advantage of avoiding the use of intravenous contrast agents, providing high-quality images, showcasing a vivid intraluminal signal intensity of the thrombus, and offering superior evaluations of neck abscesses, potential intracranial complications, and metastases, but it has several limitations in recognizing lesions in lung parenchyma. Consequently, while MRI can offer valuable insights, its utilization as a routine diagnostic tool is not commonly endorsed [9]. However, combined CT and MRI provide complementary information. The choice between CT and MRI for Lemierre syndrome hinges on the clinical context, the required information, and the individual patient’s characteristics. Frequently, a combination of both imaging modalities may be employed to ensure a thorough and comprehensive assessment.

### 2.5. What Treatment Regimens Are Most Used?

In the management of Lemierre’s syndrome, intravenous antibiotics play a pivotal role. Common empiric antibiotic regimens often involve using beta-lactam/beta-lactamase inhibitors or carbapenem coverage. A recent systematic review [23] highlighted that the most frequently employed regimen consisted of carbapenem and piperacillin/tazobactam, administered either as monotherapy or combined with metronidazole to address anaerobic activity. Additionally, combining ceftriaxone with metronidazole was noted to achieve the desired coverage of both *F. necrophorum* and oral *streptococci* [9]. The typical duration of antibiotic therapy ranges from 3 to 6 weeks [24,25]. It is imperative to highlight that the selection of antibiotics can be tailored based on the specific pathogens identified and their susceptibilities, a determination typically made through blood cultures or other relevant cultures [26]. Adjusting antibiotic regimens based on microbial profiles is crucial for optimizing treatment efficacy. In some cases, adjunctive anticoagulation may be considered as it can prevent septic emboli. However, it is important to note that optimal anticoagulation regimens for Lemierre’s syndrome need to be clearly defined [27]. Upon reviewing the literature, it was found that anticoagulation was utilized in approximately 63.7% of pediatric Lemierre’s syndrome cases [28]. Surgical drainage is sometimes required for large abscesses [29].

### 2.6. What Complications and Metastatic Foci Occur in Pediatric Lemierre’s Syndrome?

The manifestations of metastatic foci of infection in Lemierre’s syndrome may be evident at the initial presentation or emerge later in the course of the illness. Given the common involvement of the lungs, clinicians should consider Lemierre’s syndrome in the differential diagnosis for patients exhibiting signs related to septic emboli, such as dyspnea, cough, respiratory distress, and chest pain [30]. Lung abscesses typically manifest on chest X-rays as well-demarcated, thick-walled cavities with an air fluid level [31]. In children, infiltrates are a common presentation, often detectable through chest X-rays. However, a contrast-enhanced chest CT scan is recommended when there is uncertainty or when no improvement is observable after appropriate treatment. The second most frequently affected sites by septic emboli are large joints, with manifestations ranging from arthralgia to septic arthritis and, rarely, osteomyelitis and muscle abscesses [9,32]. Other potential complications encompass splenic and liver abscesses, endocarditis, pericarditis, soft tissue abscesses, and septic shock. Central nervous system (CNS) complications have been reported in 10–16% of cases but may include meningitis, subdural empyema, cerebral infarction, and cerebral venous thrombosis resulting from retrograde extension of thrombosis [5]. CT scans can reveal intracranial extension of thrombosis, even in the absence of new CNS signs. Pulmonary complications, including effusions, empyema, and septic emboli, occur more frequently in pediatric cases. Liver abscesses, endocarditis, and pericarditis may also rarely complicate the disease course [5,9].

### 2.7. What Are the Common Morbidity and Mortality Rates with Appropriate Treatment?

While the effective management of Lemierre’s syndrome has significantly reduced mortality rates, the morbidity associated with this condition remains a considerable challenge [6,9,21]. The latest meta-analysis in the medical literature made a concerted effort to ascertain the mortality rate associated with Lemierre’s syndrome [3]. This analysis included not only the calculation of a confidence interval but also a series of sensitivity analyses to ensure the robustness of the findings. The reported overall mortality rate was approximately 4%, reflecting the seriousness of the condition despite advances in medical treatment and diagnostic techniques [3]. It is critical for healthcare providers to recognize the potential severity of Lemierre’s syndrome and to manage it aggressively to minimize the risk of fatal outcomes. With prompt diagnosis and the initiation of appropriate antibiotic therapy, the acute threat to life is greatly diminished. Still, the journey to full recovery can be fraught with complications and requires extensive medical care [3,4]. Metastatic infections are a prominent source of morbidity in Lemierre’s syndrome due to septic emboli, or infected blood clots, dislodging from the site of the original infection and spreading through the bloodstream to distant organs [26]. Several secondary infections can develop, such as pneumonia [5,31], septic arthritis, osteomyelitis, or even meningitis [33,34]. Managing these infections often requires prolonged courses of targeted intravenous antibiotic therapy, which can extend hospital stays and increase the risk of hospital-acquired infections [35,36]. The need for intensive care unit (ICU) admission is indicative of the severity of this syndrome, with reports suggesting that up to two-thirds of pediatric patients with Lemierre’s syndrome require ICU care [37]. Timely and effective treatment has been instrumental in reducing the mortality rate of Lemierre’s syndrome to 6% or below [3]. The stark contrast between historical data, which reported a mortality rate nearing 90%, and current outcomes underscores the critical need for the prompt identification and treatment of Lemierre’s syndrome [2]. The continued risk of mortality, even with treatment, is attributed to several factors, including the virulence of the causative pathogen, often Fusobacterium necrophorum [29], the patient’s underlying health status, and the presence of co-infections [22] or other complications [30]. Rapidly progressing sepsis, multiorgan failure, and severe immune responses to the infection are among the critical factors that can lead to a fatal outcome despite aggressive treatment [38].

### 2.8. What Risk Factors Predispose Children to Developing Lemierre’s Syndrome?

The potential main risk factor for pediatric patients with Lemierre’s syndrome is previous oropharyngeal infection. The mechanism by which *F. necrophorum* or other microorganisms gain entry into the internal jugular vein from their initial point of infection in the oropharynx is indeed quite straightforward [26,27,28,29,30]. The lymphatic vessels responsible for draining the deeper structures of the head run parallel to this vein. Furthermore, numerous lymph nodes associated with these lymphatic vessels are situated near the internal jugular vein [27]. These anatomical relationships facilitate the dissemination of pathogens from localized infections to systemic circulation. However, additional factors that may increase the likelihood of a child developing this syndrome have been identified [39]. Among these, a recent history of viral infection, such as infectious mononucleosis, has been shown to compromise the oropharyngeal mucosa, making it potentially susceptible to bacterial invasion [21,40]. Children who are immunocompromised due to conditions such as diabetes or HIV or due to immunosuppressive therapy for other medical conditions may also have a higher risk [41,42,43]. Lesions of the oropharyngeal mucosa, which can result from an apparently benign event such as a throat swab or tonsillectomy [44], could predispose a child to develop Lemierre’s syndrome by providing a pathway for bacteria to invade the jugular vein [38,40]. Genetic predisposition may also play a role in some cases, although the specific genetic factors involved are still being studied [45]. It is critical to emphasize that Lemierre’s syndrome does not exclusively affect individuals with identifiable risk factors.

Indeed, all the risk factors often cited in relation to Lemierre’s syndrome have typically been proposed based on anecdotal evidence from isolated case reports. They have not been substantiated by statistical associations derived from methodical investigations, such as longitudinal or cross-sectional studies. Therefore, while these factors may provide clinical clues, they lack the empirical validation that would come from more rigorous scientific research. Even healthy children and adolescents without apparent predisposing conditions can develop this syndrome, although rarely [46]. This underscores the importance of being vigilant toward the syndrome’s symptoms and ensuring their early recognition in all pediatric patients.

### 2.9. How Do the Pathogenesis, Presentation, and Management Differ between Children and Adults?

Lemierre syndrome follows a similar pathogenetic trajectory in both children and adults [27]. However, the syndrome’s manifestation can differ greatly between the two groups, especially in the pediatric population under two years of age [47]. Infants and children under two years of age, who are often unable to articulate symptoms, may present with nonspecific signs such as irritability, mild fever, and poor feeding [47,48]. Such symptomatology may be misleadingly attributed to teething, minor viral infections, or gastrointestinal disorders [38]. This ambiguity in pediatric clinical presentation can significantly hinder timely diagnosis. In contrast, children over two years of age and adults usually present with more recognizable signs of infection, including a high fever, a sore throat, and neck swelling or tension [49], which may prompt healthcare providers to consider a broader differential diagnosis, including Lemierre’s syndrome. Despite these differences in presentation, the fundamental strategies for managing Lemierre’s syndrome share many similarities across age groups [50]. The length of antibiotic therapy tends to be consistent between pediatric and adult patients, generally spanning several weeks to ensure the complete eradication of the infection [51,52]. However, the specific antibiotics used and dosage may vary, with pediatricians considering the child’s age, weight, and developmental stage to optimize dosing and minimize potential adverse effects [42]. The management of Lemierre’s syndrome may require anticoagulants [28,53,54,55,56,57], though the use of such treatments is debated, particularly in infants, who require careful consideration due to their unique hemostatic system and the risk of bleeding. Supportive care measures such as hydration and pain management are tailored to the needs of the pediatric patient and are age-appropriate [13].

### 2.10. What Are the Long-Term Outcomes for Pediatric Patients?

Most pediatric patients who recover from the acute phase of Lemierre’s syndrome may expect to have favorable long-term outcomes [43,51,58]. While the majority of individuals with Lemierre’s syndrome recover without lasting effects, there is a significant minority—about 10%—who experience permanent sequelae. This is particularly concerning when taking into account the patient population often affected by Lemierre’s syndrome, which predominantly includes children and young adults. The long-term impact on this younger demographic underscores the importance of timely diagnosis, effective treatment, and thorough follow-up care for mitigating the risk of chronic complications. Following recovery, these children typically continue on a normal trajectory of growth and development, with no discernible impact from their brush with this serious illness [3,11]. Despite the generally positive prognosis, a subset of pediatric survivors of Lemierre’s syndrome, estimated at around 10%, may face persistent health issues as a result of their illness [59]. These long-term sequelae can vary in severity and may include neurological effects such as cranial nerve palsy [60,61]. Visual impairments are also among the potential long-term consequences, with some children experiencing blindness or significantly diminished visual acuity [62]. Monitoring for jugular vein recanalization and carotid stenosis is a relevant aspect of this follow-up due to their associated risks, such as chronic cerebrospinal venous insufficiency (CCSVI) [55,63,64,65]. CCSVI may lead to narrowing or blockage of the internal jugular veins, disrupted blood flow, the development of alternative venous pathways, or collateral circulation. Despite the routine administration of antibiotics, the intricate anatomy of the deep neck spaces can conceal the full scope of an infection. Therefore, when there is a significant infection involving the internal jugular veins (IJVs), a concurrent evaluation of the carotid arteries is advisable to exclude the possibility of the infection spreading to these vessels [66]. A thorough understanding of neck anatomy enables radiologists to swiftly trace the likely pathways of infection dissemination. This provides surgeons with vital information that can inform surgical planning and intervention strategies. This collaborative approach between radiologists and surgeons is essential for promptly and effectively managing deep neck infections.

## 3. Discussion

Lemierre’s syndrome, a rare but serious septic thrombophlebitis of the internal jugular vein, primarily follows an oropharyngeal infection. It is most notorious for its high morbidity and mortality rates if not promptly recognized and treated [2,3,4]. The syndrome, largely considered a historical footnote in the antibiotic era, has seen a resurgence that has been attributed to several factors, including the judicious use of antibiotics leading to increased bacterial resistance, improved detection methods, and a growing awareness among clinicians [6,7,8,9]. The syndrome predominantly affects healthy adolescents and young adults, involving a typical presentation of protracted pharyngitis followed by fever, neck pain, and swelling. The causative pathogen, *F. necrophorum*, is isolated in most cases, but the incidence of polymicrobial infections is also significant [66]. The role of imaging in Lemierre’s syndrome is pivotal, with contrast-enhanced CT of the neck serving as the gold standard for diagnosis. However, MRI is increasingly being recognized for its utility in detecting thrombosis and potential metastatic infections [10,12,16]. In the Emergency Department, the detection of IJV thrombophlebitis is common, with ultrasounds being capable of revealing the thrombus as either a hyperechoic or hypoechoic mass [6]. The application of color Doppler and spectral Doppler imaging plays a crucial role in evaluating blood flow and identifying any occlusions or irregular flow patterns indicative of a thrombus. However, it is well recognized that ultrasounds have limitations regarding their diagnostic accuracy, sensitivity, specificity, and capacity to visualize clots in difficult locations or clots that are newly formed and exhibit low echogenicity [10,11,12,13,14]. The proficiency and experience of the operator are crucial determinants of the US success in identifying IJV thrombophlebitis. Employing strategies to evaluate vein collapsibility without direct compression, such as repositioning the patient, can improve the safety and effectiveness of thrombus detection [15]. Despite these limitations, the imaging modality of choice for a definitive diagnosis remains a contrast-enhanced CT scan of the neck and chest. CT scans are particularly effective at delineating the primary site of infection and the specific features associated with it, including filling defects in the IJV and surrounding soft tissue swelling [16]. They are especially skilled at pinpointing complications like osteomyelitis, arthritis, abscesses, and septic pulmonary embolisms while providing details about their size and spread, often revealing a typical lung pattern [17]. Most cases display the “feeding vessel sign,” a unique indication of a blood vessel leading to a lesion [17]. CT also proves invaluable in atypical presentations, such as when thrombosis occurs in veins other than the IJV [18,19,20], and it is crucial in diagnosing this condition, which can have a high mortality rate [22]. In contrast, MRI offers superior soft-tissue contrast and is particularly sensitive to slow-flowing blood, which gives it an advantage in assessing neck abscesses, intracranial complications, and metastases [9]. However, MRI is less adept at identifying lung parenchymal lesions, a critical aspect given the pulmonary involvement in Lemierre syndrome. Consequently, the choice between CT and MRI depends on the specific clinical circumstances, the information needed for accurate diagnosis and treatment planning, and patient factors. Often, a combination of both imaging methods is employed to ensure a comprehensive and precise diagnostic workup, capitalizing on the strengths of each technique. Treatment regimens center around the administration of intravenous antibiotics, with the inclusion of anticoagulation therapy in some cases. However, despite the availability of potent antibiotics, mortality rates can reach up to 18%, with significant morbidity stemming from potential metastatic infections [3,33,34]. The need for a multi-week course of antibiotics and, in some cases, intensive care admission further underscores the severity of the condition [32,35]. Given these considerations, several areas require attention to improve the outcomes of patients with Lemierre’s syndrome. First, developing sensitive and specific biomarkers could allow for earlier detection, which is critical in a disease where early intervention can dramatically alter the prognosis. The current reliance on clinical presentation and imaging may not be sufficient, particularly in atypical presentations or in resource-limited settings. The phenomenon of antibiotic resistance is of particular concern in the management of Lemierre’s syndrome [36,37]. With *F. necrophorum* and other potential anaerobic pathogens showing increased resistance to common antibiotics, research into these patterns is essential for informing empirical therapy [5,9,34,38]. This must be balanced against the imperative to avoid contributing to the wider problem of antibiotic resistance, highlighting the need for refined antibiotic stewardship [39]. Anticoagulation’s role in treating internal jugular vein thrombosis secondary to Lemierre’s syndrome remains controversial. Current practices vary, with there being no consensus on the optimal regimen or duration of therapy [40,41,42]. This area is ripe for research, with the potential for randomized controlled trials to provide much-needed evidence to guide clinical practice. Indeed, considering the rarity of Lemierre syndrome, executing a clinical trial to directly compare the diagnostic accuracy of CT and MRI would be exceedingly challenging, even within a multicenter framework. The low incidence of the disease makes the collection of a substantial sample size for a randomized controlled trial highly improbable. Instead, a meta-analysis of open-label reports and observational studies may be a more practical and effective approach to gather and analyze data on this infrequent condition, allowing for the consolidation of evidence from a range of clinical settings and patient scenarios. The management of Lemierre’s syndrome also necessitates a long-term perspective on patient care. While the acute phase of the illness is the most critical, patients require follow-up to monitor for complications such as post-thrombotic syndrome or recurrence [67,68]. Longitudinal studies are necessary to better understand these long-term outcomes and to develop evidence-based guidelines for follow-up care [3,34,43]. Preventive measures against Lemierre’s syndrome, such as vaccines against *F. necrophorum*, may have a role in the future [44]. Although developing such vaccines is challenging and likely not a short-term solution, research in this area could eventually contribute to preventing the syndrome. However, it should be considered that the rarity of the disease makes it more likely that vaccine-associated complications will be more common than the disease, resulting in a negative overall balance for potential indication. A multidisciplinary approach is essential for the effective management of Lemierre’s syndrome. This approach should involve a range of specialties, including pediatrics, infectious disease, otolaryngology, and radiology, to ensure that all aspects of the disease are addressed [5]. Clinical pathways can streamline this approach, ensuring that each patient receives timely and comprehensive care. Education is another critical component in the fight against Lemierre’s syndrome. Both healthcare providers and the public must be aware of the syndrome’s signs and symptoms, as early recognition and intervention are paramount. Educational programs and campaigns can raise awareness and ensure that patients seek medical attention promptly for persistent throat infections [45]. Lastly, advocacy for increased research funding is crucial. Despite the syndrome’s potentially devastating consequences, it has not received the research attention it deserves [49,54,55]. Advocacy could bring the syndrome to the forefront of medical research agendas, facilitating the development of better diagnostic and treatment modalities. While significant progress has been made in understanding and managing Lemierre’s syndrome, there is ample room for advancement. Enhanced diagnostic protocols, antibiotic stewardship, research into adjunctive therapies, long-term patient management, prevention strategies, multidisciplinary approaches, education, and research funding are all areas that, if addressed, could improve the outcomes for patients with this serious condition [19,25]. The medical community’s commitment to these focus areas is essential to ensure that Lemierre’s syndrome is consistently detected early and managed effectively, thereby reducing its burden on both patients and healthcare systems.

## 4. Conclusions/Future Directions

Lemierre’s syndrome should be suspected in previously healthy children or young adults who have reported persistent pharyngitis followed by unilateral neck swelling or pain accompanied by fever, particularly if their symptoms evolve into an unclear septic picture following a head and neck infection. The prompt recognition of these signs is crucial for the early diagnosis and treatment of this potentially life-threatening condition. Early imaging with contrast-enhanced CT or MRI of the neck to assess for internal jugular vein thrombophlebitis is recommended when Lemierre’s syndrome is clinically suspected. Despite negative blood cultures, empiric broad-spectrum antibiotics to cover anaerobic organisms should be initiated urgently at the earliest clinical suspicion. A high degree of awareness for Lemierre’s syndrome allows for timely diagnosis and treatment, which is imperative given the disease’s high mortality rate if left untreated. Unresolved aspects in the management of Lemierre’s syndrome include determining the optimal antibiotic and anticoagulant regimen, as well as establishing the appropriate duration of treatment. Given the low incidence of Lemierre’s syndrome, there is a pressing need for multicenter randomized clinical trials and international consensus efforts. These endeavors are crucial for enhancing our understanding, refining treatment approaches, and establishing standardized clinical care for this rare condition. As the medical community continues to confront Lemierre’s syndrome, future directions should concentrate on several key areas to advance the understanding and treatment of this serious condition. Enhanced diagnostic protocols, including the development of specific biomarkers, could significantly reduce the time to diagnosis, allowing for earlier and more targeted interventions. The issue of antibiotic resistance necessitates ongoing surveillance and research into the patterns of resistance among causative organisms, ensuring that empirical treatments remain effective. This effort should be coupled with robust antibiotic stewardship programs to prevent the further development of resistance. Anticoagulation therapy, a contentious aspect of treatment, requires rigorous investigation to establish standardized guidelines. Prospective studies or randomized controlled trials could clarify its role, optimal duration, and dosage, providing evidence that is much needed to inform clinical decisions. Long-term patient management is another critical area, with the need for longitudinal studies to assess the long-term outcomes and potential late complications in pediatric patients. Such investigations would contribute to the formulation of comprehensive follow-up care protocols. Preventive strategies, while challenging, should not be overlooked. Research into vaccines targeting *F. necrophorum* and other implicated pathogens could lead to a reduction in incidence. Additionally, multidisciplinary collaboration is essential for improving patient outcomes, and efforts should be made to develop integrated clinical pathways that involve all relevant specialties from the point of diagnosis through to treatment and follow-up care. Education campaigns targeting both healthcare providers and the public are necessary to raise awareness about Lemierre’s syndrome, emphasizing the importance of the early recognition and treatment of persistent oropharyngeal infections. Lastly, advocacy for increased research funding is paramount. Given the disease’s potential severity and the gaps in knowledge, particularly regarding long-term outcomes and optimal management strategies, enhanced financing is critical to advancing the research agenda. Addressing these areas will help to ensure that Lemierre’s syndrome is consistently identified promptly and managed effectively, ultimately improving patient prognoses and reducing the healthcare burden associated with this condition.

## Data Availability

No new data were created or analyzed in this study. Data sharing is not applicable to this article.
